# A new nomogram for predicting 90-day outcomes of intravenous thrombolysis in patients with acute ischaemic stroke

**DOI:** 10.3389/fneur.2025.1512913

**Published:** 2025-02-27

**Authors:** Yingjie Zhao, Rui Zhang, Pan Li, Zhen Zhang, Huan Yu, Zhaoya Su, Yandong Xia, Aiguo Meng

**Affiliations:** Department of Clinical Laboratory, North China University of Science and Technology Affiliated Hospital, Tangshan, China

**Keywords:** acute ischemic stroke, SII, SIRI, intravenous thrombolysis, nomogram

## Abstract

**Background:**

The aim of this study was to construct and validate a new nomogram to predict the risk of poor outcome in patients with acute ischemic stroke (AIS) after intravenous thrombolytic therapy (IVT).

**Methods:**

A total of 425 patients who received IVT within 4.5 h of stroke onset were included in a retrospective study. All the patients were divided into training (70%, *n* = 298) and validation cohorts (30%, *n* = 127). Poor outcome (defined as a 90-day modified Rankin Scale score 3–5) was the primary outcome. Logistic regression was used for analysis of independent risk factors for poor outcome in patients with AIS. Nomograms of poor outcome in AIS patients were constructed using R software. Discrimination and calibration of the models were assessed using area under the receiver operating characteristic (ROC) curve (AUC) and calibration plots.

**Results:**

Multifactorial logistic regression analysis showed that SII (OR = 1.001, 95% CI: 1.000–1.002, *p* = 0.008), SIRI (OR = 1.584, 95% CI: 1.122–2.236, *p* = 0.009), NIHSS (OR = 1.101, 95% CI: 1.044–1.160, *p* < 0.001), and history of diabetes mellitus (OR = 2.582, 95% CI: 1.285–5.188, *p* = 0.008) were the independent risk factors for the occurrence of poor outcome in AIS patients. The poor outcome nomogram for AIS patients was constructed based on the above independent risk factors. The training and validation cohort AUCs of the nomogram were 0.854 (95% CI: 0.807–0.901) and 0.855 (95% CI: 0.783–0.927), respectively. The prediction models were well calibrated in both the training and validation cohorts. The net benefit of the nomograms was better when the threshold probability ranges were 4.28–66.4% and 4.01–67.8% for the training and validation cohorts, respectively.

**Conclusion:**

New nomogram includes NIHSS, SII, SIRI and diabetes as variables with the potential to predict the risk of 90-day outcomes in patients with AIS following IVT.

## 1 Introduction

Stroke, a sudden neurological disorder, is a leading cause of disability and death in adults (1). Among stroke cases, acute ischemic stroke (AIS) accounts for 60 to 80% (2). Intravenous thrombolysis with recombinant tissue-type plasminogen activator (rt-PA) within 4.5 h of onset of symptoms is the treatment of choice and significantly improves neurological function in patients (3). However, a certain percentage of patients continue to experience poor prognostic outcomes (4). Therefore, the early identification of patients at risk for poor outcome, along with timely and accurate therapeutic interventions, is crucial for improving patient recovery and outcomes (5, 6). A nomogram is a visual scoring model that utilizes biological and clinical variables to accurately calculate the probability of an individual patient’s risk for a specific clinical event (7). The chart is widely used for clinical decision-making in a wide range of conditions (8, 9). Nomograms surpass traditional scoring systems in their ability to more accurately identify patients with poor outcome, assist in selecting optimal treatment options, and enhance the quality of patient survival (10). The aim of this study was to construct a nomogram to predict the risk probability of poor outcome in AIS patients following intravenous thrombolytic therapy.

## 2 Materials and methods

### 2.1 Patients and participants

The data utilized in this study were sourced from the Hospital of North China University of Science and Technology, covering the period from June 2021 to October 2023. This retrospective cohort study included 425 patients diagnosed with acute ischemic stroke who underwent intravenous thrombolysis at our facility.

The inclusion criteria were as follows: (1) patients met the diagnostic criteria outlined in the Chinese AIS diagnostic and treatment guidelines; (2) age was ≥18 years; (3) the time from symptom onset to the administration of rt-PA intravenous thrombolysis was less than 4.5 h; (4) the modified Rankin Scale (mRS) score was ≤2 prior to the onset of the disease; and (5) patients provided signed informed consent. Patients with the following conditions were excluded from the study: (1) those who underwent intravenous thrombolysis followed by arterial thrombolysis or endovascular thrombolysis; (2) individuals with autoimmune diseases; and (3) patients with incomplete clinical data. This study received review and approval from the Institutional Research Review Board of North China University of Science and Technology Hospital.

### 2.2 Data collection and computation

Demographic characteristics of all participating patients were collected, including age, National Institutes of Health Stroke Scale (NIHSS) score, sex, and vascular risk factors such as history of alcohol consumption, hypertension, smoking, diabetes mellitus, previous stroke, hyperlipidemia, coronary heart disease, hyperhomocysteinemia, and atrial fibrillation. Clinical information gathered included systolic blood pressure, diastolic blood pressure, and blood glucose levels. Laboratory data encompassed SII, SIRI, platelet count, total cholesterol, LDL cholesterol, triglycerides, and HDL cholesterol. All blood samples used for laboratory testing were obtained from the first blood draw conducted just prior to the patient’s admission for intravenous thrombolysis. Hyperhomocysteinemia is defined as an elevated serum homocysteine concentration of more than 15 μmol/L (11). SIRI is defined as the product of neutrophil count and monocyte count divided by lymphocyte count, while SII is calculated as the product of platelet count and neutrophil count divided by lymphocyte count.

### 2.3 Outcome

The outcome was determined by the modified Rankin Score (mRs) at 90 days after thrombolysis. Poor outcome was defined as an mRs score of 3–5.

### 2.4 Thrombolysis method

Upon admission, all patients received guideline-based therapy, specifically recombinant tissue plasminogen activator, administered within 4.5 h of stroke onset. The dosage was established at 0.9 mg/kg, with a maximum limit of 90 mg. Ten percent of the total dose was administered as an intravenous bolus, followed by a continuous intravenous infusion of the remaining 90% over a duration of 60 min.

### 2.5 Statistical analysis

Data analysis and nomogram construction were conducted utilizing SPSS software version 27.0 and R software version 4.4.0. Measurement data were reported as means with standard deviations (SDs) or medians with interquartile ranges (IQRs). Group comparisons were performed using *t*-tests or Mann–Whitney *U* nonparametric tests, while count data were expressed as frequencies with percentages. Comparisons between the two groups were assessed using the *χ*^2^ test.

Variables that achieved statistical significance at *p* < 0.05 in the univariate analysis were subsequently included in the multivariate logistic regression analysis to identify independent risk factors associated with poor prognostic outcomes at 90 days. Event outcomes were assigned values of “1” for poor outcome and “0” for good outcome. Independent risk factor variables were incorporated using R software to develop a new nomogram prediction model.

## 3 Results

A total of 465 patients who received intravenous thrombolysis with rt-PA were enrolled in this study. A total of 42 patients were excluded from the study due to various reasons: 21 patients underwent endovascular thrombolysis, 6 patients had autoimmune diseases, and 15 patients had incomplete follow-up data. Ultimately, 425 patients were successfully enrolled in this study, among which 102 patients experienced poor outcome, resulting in an incidence rate of 24%.

All subjects who met the established criteria were randomly assigned to the training cohort (*n* = 298) and the validation cohort (*n* = 127) in a 7:3 ratio. The median age of participants was 66 (57, 72) years in the training cohort and 65 (59, 71) in the validation cohort. There were no significant differences between the two groups regarding age, gender, or medical history (all *p* > 0.05, Table 1).

**Table 1:** Baseline characteristics of eligible participants.

Variables	Total sample (*n* = 425)	Training set (*n* = 298)	Testing set (*n* = 127)	*p*-value
Age (years), median (IQR)	65 (58–72)	66 (57–72)	65 (59–71)	0.876
Gender (male)	295 (69.4)	206 (69.1)	89 (70.1)	0.936
Smoking, *n* (%)	178 (41.9)	121 (40.6)	57 (44.9)	0.477
Drinking, *n* (%)	110 (25.9)	79 (26.5)	31 (24.4)	0.740
Hypertension, *n* (%)	255 (60.0)	176 (59.1)	79 (62.2)	0.619
Diabetes mellitus, *n* (%)	102 (24.0)	76 (25.5)	26 (20.5)	0.323
Hyperlipidemia, *n* (%)	245 (57.6)	164 (55.0)	81 (63.9)	0.118
Atrial fibrillation, *n* (%)	30 (7.1)	20 (6.7)	10 (7.9)	0.825
Hyperhomocysteinemia, *n* (%)	114 (26.8)	77 (25.8)	37 (29.1)	0.560
Coronary heart disease, *n* (%)	75 (17.6)	60 (20.1)	15 (11.8)	0.055
History of stroke, *n* (%)	155 (36.5)	106 (35.6)	49 (38.6)	0.630
SBP (mmHg)	156.27 ± 23.69	156.40 ± 23.68	155.98 ± 23.80	0.892
DBP (mmHg)	90.03 ± 13.28	90.17 ± 13.26	89.71 ± 13.18	0.551
NIHSS (score)	6 (4–10)	6 (4–10)	6 (4–11)	0.913
Platelet (10^9^/L)	222 (176–269)	223 (176–275)	215 (176–267)	0.438
TC (mmol/L)	4.89 (4.12–5.71)	4.90 (4.09–5.79)	4.88 (4.20–5.50)	0.702
TG (mmol/L)	1.63 (1.15–2.49)	1.64 (1.12–2.44)	1.63 (1.15–2.58)	0.516
HDL (mmol/L)	1.20 (1.02–1.46)	1.25 (1.02–1.47)	1.22 (1.03–1.45)	0.691
LDL (mmol/L)	3.08 (2.44–3.63)	3.04 (2.39–3.65)	3.14 (2.52–3.63)	0.673
Blood glucose (mmol/L)	6.58 (5.69–8.47)	6.58 (5.68–8.49)	6.66 (5.77–7.89)	0.902
SII	623.33 (398.19–967.66)	625.63 (396.21–980.08)	614.42 (403.54–896.79)	0.497
SIRI	1.15 (0.78–1.89)	1.18 (0.78–1.97)	1.14 (0.76–1.80)	0.308

NHISS, National Institute Health of Stroke Scale; SBP, systolic blood pressure; DBP, diastolic blood pressure; HDL, high-density lipoprotein; LDL, low-density lipoprotein; TG, triglyceride; TC, total cholesterol; SIRI: systemic inflammation response index; SII: systemic immune-inflammation index. Data are shown as mean ± SD or median (IQR) for continuous variables and as number (percentage) for categorical variables.

Univariate analysis of the training set showed that NIHSS, SII, SIRI, age, and history of diabetes were significantly associated with poor outcome in patients with AIS. Further multifactorial logistic regression analysis confirmed that SII (OR = 1.001, 95% CI: 1.000–1.002, *p* = 0.008), SIRI (OR = 1.584, 95% CI: 1.122–2.236, *p* = 0.009), NIHSS (OR = 1.101, 95% CI: 1.044–1.160, *p* < 0.001), and history of diabetes mellitus (OR = 2.582, 95% CI: 1.285–5.188, *p* = 0.008) were identified as independent risk factors for the development of poor outcome in patients with AIS (Table 2).

**Table 2:** Parameters of poor outcome risk in AIS patients after IVT therapy based on univariable and multivariable analyses of the training dataset.

Variables	Univariable analysis			Multivariable analysis		
	OR	95% CI	*p*-value	OR	95% CI	*p*-value
Age	1.027	(1.001–1.054)	0.044	1.025	(0.993–1.057)	0.131
Gender	0.692	(0.397–1.207)	0.194			
NIHSS	1.096	(1.046–1.148)	<0.001	1.101	(1.044–1.160)	<0.001
Smoking	0.700	(0.403–1.214)	0.204			
Drinking	1.062	(0.586–1.923)	0.843			
Hypertension	0.920	(0.539–1.571)	0.760			
Diabetes mellitus	1.936	(1.092–3.434)	0.024	2.582	(1.285–5.188)	0.008
Hyperlipidemia	1.062	(0.624–1.808)	0.823			
Atrial fibrillation	1.730	(0.663–4.515)	0.263			
Hyperhomocysteinemia	0.835	(0.450–1.549)	0.567			
Coronary heart disease	1.579	(0.847–2.944)	0.151			
History of stroke	1.476	(0.860–2.535)	0.158			
SBP	1.002	(0.991–1.013)	0.715			
DBP	1.002	(0.982–1.022)	0.883			
Platelet	1.001	(0.997–1.005)	0.528			
TC	1.004	(0.823–1.225)	0.969			
TG	1.028	(0.866–1.221)	0.752			
HDL	1.591	(0.698–3.625)	0.269			
LDL	0.990	(0.765–1.281)	0.939			
Blood glucose	1.042	(0.967–1.122)	0.282			
SII	1.002	(1.001–1.003)	<0.001	1.001	(1.000–1.002)	0.008
SIRI	2.251	(1.740–2.913)	<0.001	1.584	(1.122–2.236)	0.009

CI, confidence interval; OR, odds ratio; NHISS, National Institute Health of Stroke Scale; SBP, systolic blood pressure; DBP, diastolic blood pressure; HDL, high-density lipoprotein; LDL, low-density lipoprotein; TG, triglyceride; TC, total cholesterol; SII, systemic immune-inflammation index; SIRI, systemic inflammation response index.

**Figure 1:**
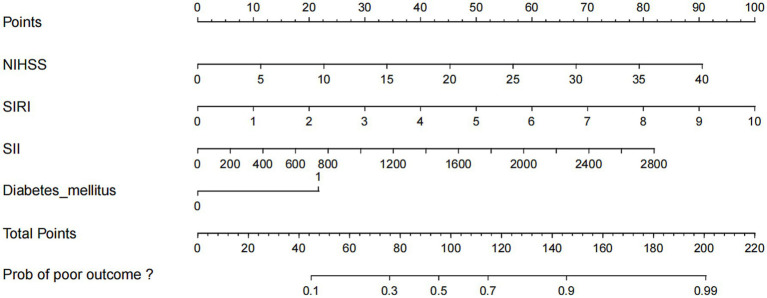
Nomogram for predicting the risk of poor outcome in AIS patients with IVT therapy. Each of the four indicator scores is aligned with the “Points” line. The four scores are then summed to produce a “Total Points” score, which can be utilized to predict the risk of poor outcome. NHISS, National Institute Health of Stroke Scale; SIRI, systemic inflammation response index; SII, systemic immune-inflammation index.

**Figure 2:**
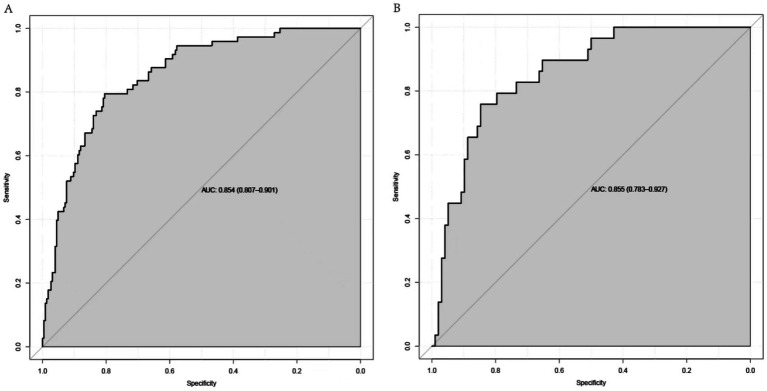
ROC of the nomogram in the **(A)** training and **(B)** testing cohorts.

**Figure 3:**
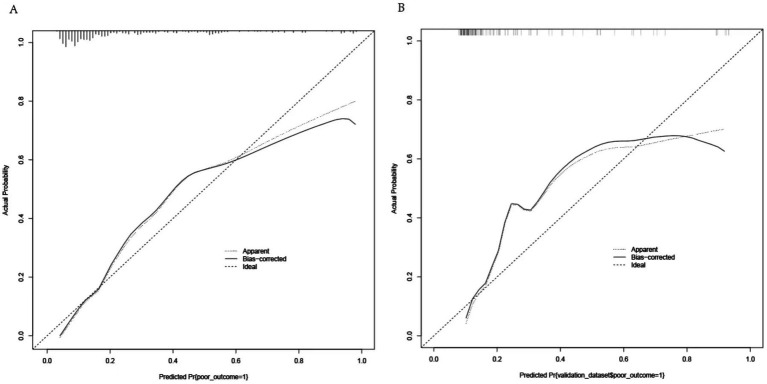
Calibration curves for the nomogram. **(A)** Training cohort: MAD = 0.042, *n* = 298. **(B)** Validation cohort: MAD = 0.067, *n* = 127. Each cohort was repeated 1,000 times.

**Figure 4:**
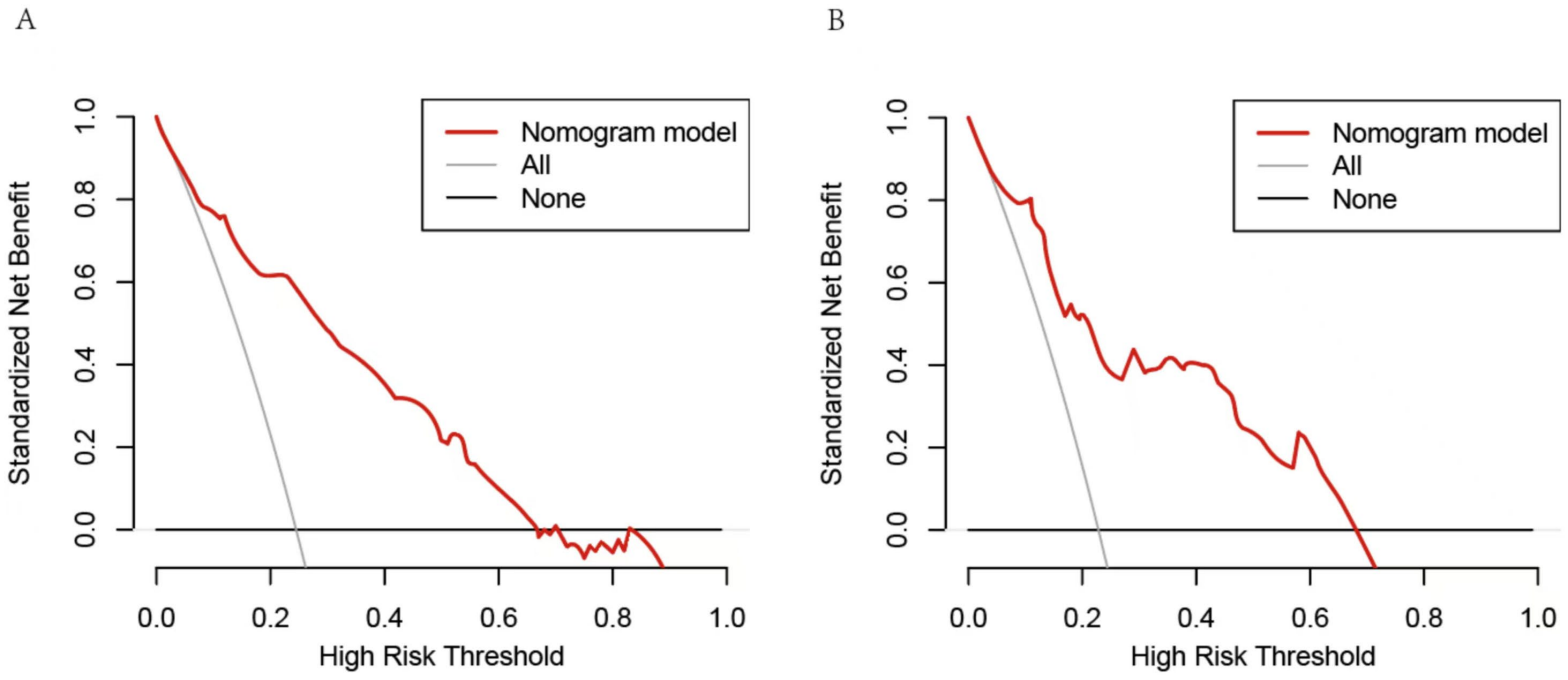
Decision curve analysis of the nomogram. **(A)** The threshold probability range for the training set was 4.28 to 66.4%. **(B)** The threshold probability range for the testing cohort was 4.01 to 67.8%. The horizontal line represents a scenario in which all factors are associated with a net benefit of zero. The dotted line means all patients who accept intravenous thrombolysis will develop poor outcome. The curves above were compared based on net benefit, represented by a backslash with a negative slope.

A nomogram was constructed to predict poor outcome following intravenous thrombolytic therapy in patients with AIS, based on independent risk factors (Figure 1). The likelihood of poor outcome for the corresponding patients was subsequently calculated. The predictive efficacy of this line plot was evaluated using area under the receiver operating characteristic curve (ROC-AUC) analysis (Figure 2; Table 3). The AUC values for the training and validation cohorts were 0.854 (95% CI: 0.807–0.901) and 0.855 (95% CI: 0.783–0.927), respectively. Calibration plots indicated that the predicted values for both the training and validation cohorts generally aligned with the actual observed values (Figure 3). To further validate the predictive ability of this nomogram regarding the incidence of poor outcome, we employed a decision curve analysis. As illustrated in Figure 4, the net benefit of utilizing the nomogram was found to be relatively high within the threshold probability ranges of 4.28 to 66.4% for the training cohort and 4.01 to 67.8% for the validation cohort.

**Table 3:** ROC parameters for the nomogram for each cohort.

	AUC	95% CI	Sensitivity (%)	Specificity (%)
Training cohort	0.854	0.807–0.901	0.795	0.804
Validation cohort	0.855	0.783–0.927	0.759	0.847

## 4 Discussion

This study included a total of 425 patients with acute ischemic stroke (AIS) who were treated with intravenous thrombolysis. Among these patients, 102 (24.0%) exhibited a poor outcome, indicating a high probability of adverse outcomes in AIS patients. Furthermore, the existing prediction models appear inadequate for accurately assessing patient outcome. Consequently, there is an urgent need for the development of new prediction models to assist clinicians in providing precise risk assessments and improving patient outcomes.

Our study identified that the systemic immune-inflammation index (SII), systemic inflammation response index (SIRI), National Institutes of Health Stroke Scale (NIHSS) score, and a history of diabetes were independent risk factors for poor short-term outcome following intravenous thrombolytic therapy in patients with acute ischemic stroke (AIS). Utilizing these independent risk factors, we developed and validated a nomogram prediction model aimed at forecasting poor outcome after intravenous thrombolytic therapy in AIS patients. Among these, SII and SIRI serve as stable markers of the inflammatory response and can be easily obtained through blood cell counts, making them both practical and reproducible. Originally proposed to be associated with poor outcomes in cancer patients (12, 13), SII and SIRI incorporate a composite index of three distinct leukocyte and platelet subpopulations, offering new insights by integrating the interplay of platelet increase, inflammation, and immunity (14, 15). As novel indicators of inflammation, SII and SIRI are linked to the inflammatory response and secondary brain damage in patients with AIS (16). Following the onset of cerebral ischemia, inflammatory cells respond rapidly, with neutrophils being the first to appear in the penumbra and infarct core (17). Once activated, neutrophils exacerbate brain edema and damage the blood-brain barrier by releasing pro-inflammatory mediators and metalloproteinases. Concurrently, additional monocytes migrate to the ischemic brain tissue, further worsening cerebral ischemia-reperfusion injury (18). An animal experiment showed that necrotic platelets interact with neutrophils during reperfusion, and rapid platelet exposure is extremely critical for inducing CypD-mediated platelet necrosis and necrosis-dependent brain injury (19). In contrast, lymphocytes primarily serve a protective function, capable of modulating the inflammatory response following a stroke, mitigating the disruption of the blood–brain barrier, and facilitating the recovery of neurological function (20). Furthermore, previous studies have indicated that elevated SII and SIRI can serve as predictors for post-stroke cognitive impairment in patients (21, 22). Additionally, research has demonstrated that SIRI and SII values correlate with a poor 90-day outcome in patients with AIS who have undergone intravenous thrombolysis, aligning with the findings of this study (23, 24).

This study demonstrates that the NIHSS score is an independent risk factor for poor 90-day outcome in patients with AIS, aligning with findings from previous research (25, 26). The NIHSS score serves as an indicator of disease severity in AIS patients; an elevated score suggests greater vascular occlusion or compromised collateral circulation capacity (27). Furthermore, the NIHSS score can predict the location and extent of infarction in stroke patients (28). Research indicates that the baseline NIHSS score also predicts post-thrombolytic hemorrhagic transformation in patients undergoing rt-PA intravenous thrombolysis (29, 30). In this study, a history of diabetes emerged as an independent risk factor for poor outcome following intravenous thrombolysis in AIS patients, with those having diabetes being 2.582 times more likely to experience poor outcomes compared to their non-diabetic counterparts. Tang et al. demonstrated that diabetes is an independent predictor of early neurologic improvement after IVT and a risk factor for incomplete recanalization at 24 h post-treatment (31). Additionally, previous studies have indicated that stroke patients with diabetes mellitus exhibit higher rates of disability and mortality following hospital discharge (32).

In the present study, novel inflammatory markers SII and SIRI were incorporated into the Short-term Adverse Outcome Scale for patients with AIS. Independent risk factors, including the NIHSS score and a history of diabetes mellitus, were also integrated into the nomogram to explore the risk of poor outcomes in AIS patients following intravenous thrombolysis in a comprehensive and cohesive manner. Almost all healthcare organizations, even those in economically disadvantaged areas, have these predictors readily available. Furthermore, validation of the nomogram indicated that the predictive model exhibited good differentiation and calibration, suggesting it may serve as a reliable tool for predicting the risk of poor outcomes in AIS patients treated with rt-PA. For patients identified by our nomogram as being at high risk for poor outcomes following thrombolysis, clinicians may consider alternative treatments to enhance clinical benefits. However, this study has certain limitations: first, it is a retrospective analysis; second, it is a single-center study, and its results have not been fully validated in an external cohort. Future prospective multicenter studies are necessary. More importantly, external validation in other cohorts is still needed before formal routine clinical practice can be established. This will provide a more reliable scientific basis for the therapeutic and prognostic assessment of patients with AIS.

## 5 Conclusion

Our study proposes a novel and practical nomogram that incorporates SII, SIRI, the NIHSS score, and a history of diabetes to effectively predict the probability of an unfavorable outcome at 90-day following intravenous thrombolysis in patients with ischemic stroke.

## Data Availability

The raw data supporting the conclusions of this article will be made available by the authors, without undue reservation.
